# Exploring the multifaceted bioactivities of *Lavandula pinnata L.* essential oil: promising pharmacological activities

**DOI:** 10.3389/fchem.2024.1383731

**Published:** 2024-04-08

**Authors:** Mounir Haddou, Amine Elbouzidi, Mohamed Taibi, Abdellah Baraich, El Hassania Loukili, Reda Bellaouchi, Ennouaamane Saalaoui, Abdeslam Asehraou, Ahmad Mohammad Salamatullah, Mohammed Bourhia, Hiba-Allah Nafidi, Mohamed Addi, Bouchra El Guerrouj, Khalid Chaabane

**Affiliations:** ^1^ Laboratoire d’Amélioration des Productions Agricoles, Biotechnologie et Environnement (LAPABE), Faculté des Sciences, Université Mohammed Premier, Oujda, Morocco; ^2^ Centre de l’Oriental des Sciences et Technologies de l’Eau et de l’Environnement (COSTEE), Université Mohammed Premier, Oujda, Morocco; ^3^ Euro-Mediterranean University of Fes (UEMF), Fes, Morocco; ^4^ Laboratory of Bioresources, Biotechnology, Ethnopharmacology and Health, Faculty of Sciences, Mohammed First University, Oujda, Morocco; ^5^ Department of Food Science and Nutrition, College of Food and Agricultural Sciences, King Saud University, Riyadh, Saudi Arabia; ^6^ Laboratory of Biotechnology and Natural Resources Valorization, Faculty of Sciences, Ibn Zohr University, Agadir, Morocco; ^7^ Department of Food Science, Faculty of Agricultural and Food Sciences, Laval University, Quebec City, QC, Canada

**Keywords:** anti-cancer, anti-diabetic, anti-gout, antibacterial, antifungal, dermatoprotective, hydrodistillation, *Lavandula pinnata*

## Abstract

**Introduction:** This study investigates the biological activities of *Lavandula pinnata* essential oil (LPEO), an endemic lavender species from the Canary Islands, traditionally used in treating various ailments.

**Methods:** LPEO was extracted by hydrodistillation and analyzed using GC-MS. Antioxidant activity was assessed by DPPH radical scavenging and total antioxidant capacity assays. Antimicrobial activity was evaluated by disc diffusion, MIC, MBC, and MFC determination against bacterial (*Staphylococcus aureus, Micrococcus luteus, Escherichia coli, Pseudomonas aeruginosa*) and fungal (*Candida glabrata, Rhodotorula glutinis, Aspergillus niger, Penicillium digitatum*) strains. Antidiabetic and anti-gout potential were investigated through α-amylase, α-glucosidase, and xanthine oxidase inhibition assays. Antityrosinase activity was determined using a modified dopachrome method. Cytotoxicity was assessed by MTT assay against breast (MCF-7, MDA-MB-468), liver (HepG2), colon (HCT-15) cancer cells, and normal cells (PBMCs).

**Results and discussion:** LPEO exhibits potent antiradical activity (IC50 = 148.33 ± 2.48 μg/mL) and significant antioxidant capacity (TAC = 171.56 ± 2.34 μg AA/mg of EO). It demonstrates notable antibacterial activity against four strains (*Staphylococcus aureus, Micrococcus luteus, Escherichia coli,* and *Pseudomonas aeruginosa*) with inhibition zones ranging from 18.70 ± 0.30 mm to 29.20 ± 0.30 mm, along with relatively low MIC and MBC values. LPEO displays significant antifungal activity against four strains (*Candida glabrata, Rhodotorula glutinis, Aspergillus niger,* and *Penicillium digitatum*) with a fungicidal effect at 1 mg/mL, surpassing the positive control (cycloheximide), and MIC and MFC values indicating a fungicidal effect. It exhibits substantial inhibition of xanthine oxidase enzyme (IC50 = 26.48 ± 0.90 μg/mL), comparable to allopurinol, and marked inhibitory effects on α-amylase (IC50 = 31.56 ± 0.46 μg/mL) and α-glucosidase (IC50 = 58.47 ± 2.35 μg/mL) enzymes.The enzyme tyrosinase is inhibited by LPEO (IC50 = 29.11 ± 0.08 mg/mL). LPEO displays moderate cytotoxic activity against breast, liver, and colon cancer cells, with low toxicity towards normal cells (PBMC). LPEO exhibits greater selectivity than cisplatin for breast (MCF-7) and colon (HCT-15) cancer cells but lower selectivity for liver (HepG2) and metastatic breast (MDA-MB-468) cancer cells. These findings suggest the potential of LPEO as an antioxidant, antimicrobial, anti-gout, antidiabetic, and anticancer agent.

## Introduction

Aromatic and medicinal plants (AMPs) have caught the attention of researchers due to their rich bioactive compound content, making them valuable for drug development and natural product exploration (Al-Mijalli et al., 2023). Essential oils (EOs) are particularly noteworthy among these compounds due to their chemical diversity and wide range of biological effects. EOs are volatile fractions extracted from aromatic plants during secondary metabolism and consist of various chemical classes, including esters of fatty acids, mono- and sesquiterpenes, phenylpropanoids, alcohols, aldehydes, and sometimes aliphatic hydrocarbons (de Oliveira et al., 2018). With these constituents, EOs possess numerous biological properties, such as antioxidant, antimicrobial, anti-diabetic, anti-inflammatory, and anticancer activities (Raut and Karuppayil, 2014). Throughout history, EOs have been utilized in therapeutic, cosmetic, culinary, and fragrance applications (Irshad et al., 2020). They can act as scavengers of free radicals or modulators of antioxidant enzyme expression (Joshi, 2014). Additionally, EOs can inhibit the growth of microorganisms, such as bacteria and fungi, by interfering with the cell membrane, protein synthesis, enzymatic activity, or energy metabolism (Knobloch et al., 1989). They can also modulate the inflammatory response by interfering with the signaling pathways involved in the production of pro-inflammatory mediators (Firmino et al., 2021). Moreover, certain constituents of EOs have significant anticancer activities, which may enhance conventional chemotherapy and radiotherapy by activating cell death and affecting cancer cell membrane potential (Lesgards et al., 2014).

*Lavandula pinnata L.* f. (syn L. pinnata Lundmark.), commonly known as Fern Leaf Lavender, is a native species of the Canary Islands. This hardy plant can withstand drought and hot weather, and it belongs to the *Lavandula* genus (Argentieri et al., 2016). The entire plant is covered in short white hairs, giving it a silvery, felt-like appearance. It consistently blooms from late spring to summer, producing single or triple-headed flower spikes on stalks measuring 8–14 inches (20–35 cm) in length. Traditionally, it has been used to treat various conditions, including skin and respiratory infections (Argentieri et al., 2016).

The aim of this study is to comprehensively evaluate the biological efficacy of *L. pinnata* essential oil (LPEO) and its primary constituents using a combination of *in vitro* methods. The essential oil’s chemical composition was determined by gas chromatography-mass spectrometry (GC-MS). The antioxidant activity of LPEO was assessed using the 2,2-diphenyl-1-picrylhydrazyl (DPPH) assay and the total antioxidant capacity (TAC) method. The antibacterial activity of LPEO was tested against Gram-positive bacteria strains (*Staphylococcus aureus* and *Micrococcus luteus*) and Gram-negative bacteria strains (*Escherichia coli* and *Pseudomonas aeruginosa*). The antifungal activity of LPEO was evaluated against two yeast species (*Candida glabrata* and *Rhodotorula glutinis*) and two mold species (*Aspergillus niger* and *Penicillium digitatum*). The cytotoxicity of LPEO was measured using the MTT assay on four human cancer cell lines (MCF-7, MDA-MB-468, HepG2, and HCT-15). These experiments were designed and conducted to gain a holistic and nuanced understanding of the multifaceted biological activity of LPEO. The primary objective of this study is to explore the potential applications of LPEO as a natural source of bioactive compounds for various purposes.

## Materials and methods

### Plant material

The plants used in this study were harvested in Ain Sfa (N 34° 46′42.36″, W 2° 9′20.043″), a rural commune located in the Oujda-Angad prefecture, in the Oriental region of Morocco, in October 2023. Throughout this period, the average monthly temperature was recorded at approximately 17.9°C, with minimum temperatures of 12°C and maximum temperatures reaching 25°C. After cultivation, these botanical specimens were transported to the Faculty of Sciences at the University Mohammed Premier Oujda (Morocco) for precise taxonomic identification. A voucher specimen number CLP-1 was deposited in the same faculty. The leaves and flowers of the plants were deliberately dried in shaded conditions to prepare them for the subsequent extraction of the essential oil.

### Essential oil extraction

LPEO was extracted by hydrodistillation, according to the method described by Guerrouj et al. (Guerrouj et al., 2023). A modified Clevenger device was used, consisting of a 2 L flask, a water-cooled condenser, and a graduated separator. 100 g of dried *L. pinnata* aerial parts, previously grounded with a mortar, were weighed. Then, 1,000 mL of distilled water was added to the flask, followed by the introduction of the plant material. The device was heated on a magnetic stirrer at a temperature of 100°C for 3 h. The steam entrained the essential oil, which condensed in the condenser and separated from the water in the separator. EO was collected in a flask. The extraction yield was calculated as a percentage relative to the initial mass of the plant.

### Qualitative and semi-quantitative analysis of LPEO

A gas chromatograph coupled with a mass spectrometer was used to identify and separate the compounds of the LPEO. The specific system used was a Shimadzu GC with a QP2010 MS. The capillary column utilized was a BPX25, which was coated with 95% dimethyl diphenylpolysiloxane. This column had a length of 30 m, an internal diameter of 0.25 mm, and a film thickness of 0.25 µm. Pure helium with a purity of 99.99% was used as the carrier gas, and it maintained a constant flow rate of 3 mL per minute. The experimental conditions were as follows: the injection temperature, ion source temperature, and interface temperature were all set at 250°C. The column oven remained at a temperature of 50°C for 1 min at the beginning. The sample components were ionized by electron impact (EI) at 70 eV. The mass of the ions was analyzed within the range of 40–300 m/z. The essential oil samples were introduced into the chamber at a volume of 1 L and diluted with an appropriate solvent. Then, 1 µL of the prepared essential oil was injected into the system using the split mode, with a split ratio of 90:1. Three evaluations were conducted for each sample to ensure the accuracy and reproducibility of the results. The identification of the compounds in the EO was accomplished by comparing their retention times and mass spectra with standards and references available in the NIST database. Finally, the Laboratory Solutions software (v2.5) was employed to collect and analyze the data.

### Tests of the antioxidant activity

In order to evaluate the antioxidant activity of LPEO, two distinct methods were used, namely, the 2,2-diphenyl-1-picrylhydrazyl (DPPH) radical scavenging test and the Total Antioxidant Capacity (TAC). The evaluation of the antioxidant activity by the DPPH radical scavenging test followed the protocols established by Elbouzidi et al. (2023), and Taibi et al. (2023). The DPPH solution was prepared by dissolving 2 mg of DPPH in 100 mL of methanol. Various concentrations of LPEO, ranging from 5 to 500 μg/mL, were prepared and added to the methanol solution containing DPPH. After incubation at room temperature for 30 min, the absorbance was quantified at a wavelength of 515 nm with respect to a control sample. The percentage of DPPH radical scavenging activity (RSA) was determined according to the established formula and the determination of the IC_50_ value was achieved through the construction of a graph correlating the percentage inhibition with the concentrations of the extract. As a reference, ascorbic acid was employed as the positive control in this experiment (Roubi et al., 2023).

$$\text{RSA }\left(\%\right)=\left[\left(\frac{A_{\text{blank}}\;-A_{\text{sample}}}{A_{\text{blank}}}\right)\right]\times 100$$

Where A_blank_ represents the absorbance of the control reaction (all reagents present except for the extract) and A_sample_ represents the absorbance of the extract at different concentrations.

The TAC of LPEO was assessed by using the phosphomolybdate method following the protocols by El Guerrouj et al. (Guerrouj et al., 2023). A sample or extract mixture with the reactive solution was heated at 95°C for 90 min. The mixture was then cooled down to room temperature and the absorbance was measured at 695 nm. The total antioxidant capacity was determined using a standard curve with different concentrations of ascorbic acid standards. The results were expressed in terms of ascorbic acid equivalents (AA). To ensure reliability, all experiments were performed in triplicate.

### Antimicrobial activity tests of LPEO

#### Disc diffusion method

Antibacterial and antifungal activity of LPEO was evaluated using the disc diffusion method as described by Alderman and Smith (2001). Four bacterial strains, including two Gram-positive (*S. aureus* (ATCC 19117™) and *M. luteus* (LB 14110)) and two Gram-negative (*E. coli* (ATCC 10536™) and *P. aeruginosa* (ATCC 15442™)), as well as four fungal strains, including two yeasts (*C. glabrata,* and *R. glutinis*) and two molds (*A. niger,* and *P. digitatum*), were used as test microorganisms. The bacteria were grown on a solid Mueller-Hinton agar medium, while the fungi were grown on a PDA (potato dextrose agar) medium. Sterile paper discs, impregnated with 15 µL of the LPEO, were placed on the surface of the media seeded with the microorganisms. Gentamicin (1 mg/mL) and cycloheximide (1 mg/mL) were used as positive controls respectively for bacteria and fungi. The Petri dishes were incubated at 37°C for 24 h for bacteria and at 25°C for 48 h for fungi. The zones of inhibition of microbial growth were measured around the disks and expressed in millimetres.

#### Determination of the MIC

The minimum inhibitory concentration (MIC) of the LPEO was determined by the 96-well culture plate method as described by Remmal et al. (1993). A Mueller-Hinton culture medium containing 0.15% agar and a PDA culture medium containing 0.15% agar were used to perform dilution series with concentrations ranging from 16% to 0.0015% of essential oil. The microplates were seeded with the bacterial and fungal strains to be tested and incubated at specific temperatures (37°C for 24 h for bacteria, 25°C for 48 h for fungi). Microbial growth was assessed by the addition of resazurin reagent, which changes color depending on the presence or absence of cellular metabolism (Lekbach et al., 2018). Positive controls, (gentamicin for bacteria and cycloheximide for fungi), were included to compare the effectiveness of the tested EO. Each experiment was performed in triplicate to ensure the reliability of the results.

#### Determination of MBC and MFC

To determine both the minimum bactericidal concentration (MBC) and the minimum fungicidal concentration (MFC), the protocol described by Thosard et *al.* (Thosar et al., 2013) was followed. Samples of 3 µL were taken from the wells where resazurin was not reduced by metabolically active cells and remained blue. These samples were then transferred to Petri dishes containing culture media (MHA for bacteria and a YEG culture medium for fungi). The dishes were then incubated at specific temperatures: 37°C for 24 h for bacteria, 25°C for 48 h for yeasts, and 72 h for molds for the MFC. After the incubation periods, minimum concentrations were established as those of LPEO causing no observable microbial growth.

### Antidiabetic and anti-gout activity/α-amylase inhibition, α-glucosidase inhibition, and xanthine oxidase assays

In this exploration of the potential antidiabetic properties inherent in LPEO, this study embarks on a comprehensive assessment integrating three pivotal assays. The α-amylase, and α-glucosidase inhibition assays serve as foundational components, meticulously delving into LPEO’s inhibitory effects on key enzymes central to glucose metabolism (Yin et al., 2014; Agarwal and Gupta, 2016; Minh et al., 2019), For the α-amylase assay, the EO undergo pre-incubation with α-amylase solution at 1 U/mL for 10 min at 37°C. The reaction initiates with the addition of 30 µL of soluble starch (0.5% in deionized water), followed by a 6-min incubation at 37°C. The reaction is then terminated with 20 µL of hydrochloric acid (1 M) and 120 µL of a 0.25 mM iodine solution. Absorbance at 565 nm is measured using a Multiskan™ Microplate Spectrophotometer. The inhibitory activity of LPEO on α-amylase was quantified as the inhibition percentage, calculated using the following formula:

$$\text{Inhibition }\left(\%\right)=\frac{\left(A-\text{Abs}\;N\right)}{\left(B-\text{Abs}\;N\right)}\times 100$$

With, A represents the absorbance of the EO, B is the absorbance of the reaction without the enzyme, and Abs N, is the absorbance of the negative control. A commercially available diabetes inhibitor, acarbose, served as a positive reference in this study.

Simultaneously, the α-glucosidase assay utilizes a methanolic stock solution mixed with 0.1 M potassium phosphate buffer and α-glucosidase enzyme solution (0.5 U/mL) for a 6-min incubation at 25°C. The reaction includes a subsequent addition of 20 µL of 5 mM *p*-nitrophenyl-α-D-glucopyranoside substrate, followed by an 8-min incubation. The reaction was concluded by adding 100 µL of 0.2 M Na_2_CO_3_, and absorbance was recorded at 405 nm (Yin et al., 2014). The inhibition percentage was calculated using the formula:

$$\text{Inhibition }\left(\%\right)=\left(1-\frac{\text{As}}{\text{Ac}}\right)\times 100$$

As: the absorbance of the isolated compound, Ac: the absorbance of acarbose (positive controls). This methodology provides a quantitative measure of the inhibitory effect of LPEO on α-glucosidase, shedding light on its potential role in modulating glucose metabolism.

Conducted under aerobic conditions, the XO assay, commonly employed for its relevance in anti-gout testing, critically explores LPEO’s inhibitory potential on xanthine oxidase (Nguyen et al., 2004). The reaction begins with the addition of 60 mL of substrate solution (150 mM xanthine in the same buffer) and incubation at 25 °C for 30 min (Nguyen et al., 2004). The reaction is halted with 25 mL of 1 N HCl, and absorbance at 290 nm is measured using a Perkin-Elmer HTS-7000 Bio-Assay Reader.

### Dermatoprotective activity: tyrosinase inhibition assay

To assess the dermatoprotective potential of LPEO, we employed a modified version of the method described by Bouyahya et al. (2019); Bouyahya et al. (2021) to evaluate tyrosinase inhibitory activity. In a nutshell, 25 μL of the LPEO sample was combined with 100 μL of tyrosinase solution (333 U/mL, 50 mM phosphate buffer, pH 6.5) and incubated at 37°C for 10 min. Following this, 300 μL of L-DOPA (5 mM) was added, and the mixture underwent a 30-min incubation at 37°C. Absorbance readings were then taken at 510 nm using a spectrophotometer. Tyrosinase inhibition levels were computed at LPEO concentrations of 40, 60, 120, and 160 μg/mL, and the IC_50_ values were determined. Quercetin was employed as the positive control in this experimental (El Hachlafi et al., 2024).

### Anti-cancer activity

#### Cell viability by MTT assay

According to the procedure outlined in (Chaudhary et al., 2015; Elbouzidi et al., 2022), the MTT test was used to assess if LPEO suppressed cancer cell growth. Exponentially multiplying MCF-7, MDA-MB-468, HepG2, and HCT-15 cells were seeded into 96-well plates (10^4^ cells/well in 100 µL of medium) and allowed to adhere for 24 h. LPEO were serially diluted with medium after being solubilized in 0.1% DMSO to reach acceptable concentrations. LPEO were applied to cells at a variety of dosages, and they were then incubated for 72 h. Cells in the control group only received media containing 0.1% DMSO. 200 μL of culture medium was used in place of the test compound media, and 20 µL of MTT reagent (5 mg/mL MTT in PBS) was added before incubation at 37°C for 4 h. The medium was taken out and 100 L of DMSO was added before a microplate reader (Synergy HT Multi-Detection microplate reader, Bio-Tek, Winooski, VT, USA) measured absorbance at 540 nm and calculated % viability (Mosmann, 1983).

$$\text{Cell }\text{viability }\left(\%\right)=100-\;\left[\left(\frac{A_{0}-A_{t}}{A_{0}}\right)\times 100\right]$$

Where At is the absorbance of cells treated with LPEO at various doses, and Ao is the absorbance of cells treated with 0.1% DMSO media. The negative control was DMSO in medium at a final concentration of 0.1% (*v/v*). Triplicates of each treatment were carried out. The standard was doxorubicin. Using dose-response inhibition curves in Graph Pad Prism 8.01, IC_50_ values were determined. PBMCs were extracted from human blood samples by Ficollhypaque density centrifugation in accordance with the manufacturer’s instructions (Capricorn Scientific). The identical settings and concentrations that were previously described for tumor cells were used to investigate the cytotoxic impact.

## Results and discussion

### Yield and phytochemical composition of the LPEO

**FIGURE 1:**
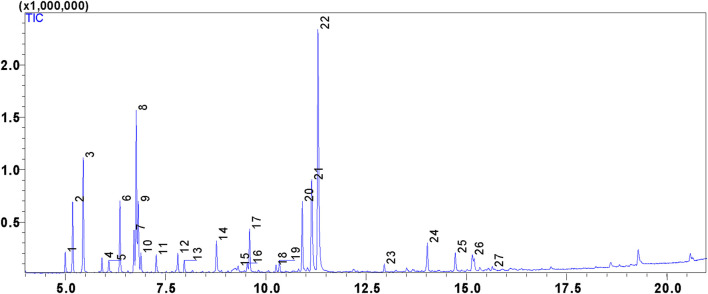
GC-MS chromatogram of the chemical composition of LPEO.

The hydrodistillation of the aerial part of *L. pinnata* yielded a return of 0.45% ± 0.03% (*w/w*). This yield can vary depending on climatic conditions, the developmental stage of the plant, and the plant material used (Mehalaine and Chenchouni, 2021; DO TRICOMA and DOS REBENTOS, 2022). The analysis by gas chromatography coupled with mass spectrometry (GC-MS) of the LPEO reveals a diversified chemical composition, comprising 27 compounds (Figure 1; Table 1), with the major compounds being: carvacrol (24.91%), D-limonene (11.28%), thymol (9.33%), camphene (7.88%). These compounds belong to the classes of monoterpenes and phenols, which are known for their antimicrobial, antioxidant, anti-inflammatory, and analgesic properties (Nagoor Meeran et al., 2017; de Alvarenga et al., 2023). The observed chemical profile aligns with the research of Argentieri et al. (2016) (Argentieri et al., 2016), who similarly identified carvacrol as the principal compound in LPEO. However, this composition diverges from the findings of Cristina Figueiredo et al. (Cristina Figueiredo et al., 1995), who analyzed essential oils of *L. pinnata* from Portugal, reporting *p*-phellandrene as the predominant component. Such disparities in chemical composition can be elucidated by the genotypic and phenotypic diversity inherent in the plant species, in conjunction with the influence of environmental factors and cultivation conditions (Tak et al., 2004; Vaičiulytė et al., 2017).

**TABLE 1:** Chemical composition of the studied *Lavandula pinnata* essential oil.

No.	Compounds	RT (min)	% Area
1	3-thujene	4.997	1.26
2	α-pinene	5.183	4.10
3	Camphene	5.445	7.88
4	β-pinene	5.913	1.12
5	β-myrcene	6.085	1.15
6	3*-*carene	6.361	4.78
7	β-cymene	6.710	2.74
8	D-limonene	6.769	11.28
9	Eucalyptol	6.819	4.54
10	2,2,6-trimethyl-cyclohexanone	6.884	1.40
11	γ-terpinene	7.267	1.29
12	Fenchone	7.802	1.60
13	3,7-dimethyl-1,6-octadien-3-ol	7.965	0.96
14	Camphor	8.767	2.65
15	4-terpinenol	9.305	0.68
16	*p*-menth-1-en-8-ol	9.536	0.73
17	*p*-methoxy-β-methylstyrene	9.592	3.15
18	Pulegone	10.252	0.58
19	3,7-dimethyl-acetate 1,6-octadien-3-ol	10.339	1.03
20	Bornyl acetate	10.907	5.06
21	Thymol	11.141	9.33
22	Carvacrol	11.298	24.91
23	Caryophyllene	12.949	0.66
24	1-methyl-4-(5-methyl-1-methylene-4-hexenyl)-, cyclohexene	14.025	2.67
25	3,7,11-trimethyl-1,6,10-dodecatrien-3-ol	14.721	1.91
26	Spathulenol	15.149	1.82
27	Selina-6-en-4-ol	15.647	0.72

### Antioxidant activity of LPEO

The antioxidant activity of LPEO was assessed through two distinct methodologies, namely, the 2,2-diphenyl-1-picrylhydrazyl (DPPH) radical scavenging assay and the determination of total antioxidant capacity (TAC). The resultant findings are meticulously tabulated in Table 2. The concentration of LPEO required to inhibit 50% of the DPPH radical is 148.33 ± 2.48 μg/mL, which shows that the EO has a good antiradical activity. The TAC of LPEO is 171.56 ± 2.34 μg of ascorbic acid per mg of essential oil, which indicates that the EO has a notable antioxidant capacity. Although the specific data on *L. pinnata* are limited in the literature, interesting conclusions can be drawn from research on other species of the genus *Lavandula*. For example, a study conducted by Messaoud et *al*. (Messaoud et al., 2012) on three species, *Lavandula coronopifolia, Lavandula multifida,* and *L. stoechas*, demonstrated that these plants have a significant antioxidant activity, in agreement with the results of the study conducted on *Lavandula dentata* by El Abdali et *al*. (El Abdali et al., 2022). The chemical composition of LPEO could be responsible for its observed antioxidant activity. These results open the way for further research on the potential use of the plant in question in the field of natural antioxidants.

**TABLE 2:** Assessement of the antioxidant activity of LPEO.

EO/Reference	Antioxidant activity	
	TAC (μg AA/mg^a^ LPEO)	DPPH (μg/mL)
LPEO	171.56 ± 2.34	148.33 ± 2.48
Ascorbic acid	-	125.23 ± 3.56

^a^ Micrograms of ascorbic acid equivalent per milligram of sample.

### Antimicrobial activity

The LPEO manifests noteworthy antimicrobial properties against a spectrum of bacterial and fungal strains (Tables 3, 4). In the context of its antibacterial activity, discernible inhibition zones were observed, surpassing the values of the positive control (Gentamicin) at 1 mg/mL. The results of Minimum Inhibitory Concentration (MIC) assays reveal conspicuously low concentrations, indicative of a substantively effective hindrance of bacterial growth. Additionally, Minimum Bactericidal Concentration (MBC) determinations affirm a bactericidal impact at relatively modest concentrations. These findings underscore a significant antibacterial efficacy, particularly evidenced by inhibition zones measuring 29.20 ± 0.30 mm for *S. aureus*, 26.50 ± 0.50 mm for *M. luteus*, 24.30 ± 0.40 mm for *E. coli*, and 18.70 ± 0.30 mm for *P. aeruginosa* (Supplementary Figures S1, S2). Correspondingly, MIC values for these strains are notably low: 0.0625% (*v/v*) for *S. aureus*, 0.125% (*v/v*) for *M. luteus*, 0.5% (*v/v*) for *E. coli*, and 1% (*v/v*) for *P. aeruginosa*. MBC values corroborate a bactericidal effect at relatively restrained concentrations: 0.25% (*v/v*) for *S. aureus*, 1% (*v/v*) for *M. luteus*, 2% (*v/v*) for *E. coli*, and 4% (*v/v*) for *P. aeruginosa*. Concomitantly, the EO demonstrates a remarkable antifungal activity, characterized by expansive inhibition zones surpassing the effects of the positive control (Cycloheximide) at 1 mg/mL. MIC values underscore a substantial capacity to impede fungal growth: 1% (*v/v*) for *P. digitatum*, 1% (*v/v*) for *A. niger*, 0.5% (*v/v*) for *C. glabrata*, and 2% (*v/v*) for *R. glutinis*. MBC values corroborate a fungicidal effect at relatively moderate concentrations: 4% (*v/v*) for *P. digitatum*, 0.25% (*v/v*) for *A. niger*, 2% (*v/v*) for *C. glabrata*, and 4% (*v/v*) for *R. glutinis*.

**TABLE 3:** Evaluation of the antibacterial activity of LPEO.

Bacterial strain	*S. aureus*	*M. luteus*	*E. coli*	*P. aeruginosa*
15 µL^a^ of Essential oil, IZ^b^	29.20 ± 0.30	26.50 ± 0.50	24.30 ± 0.40	18.70 ± 0.30
15 µL^a^ Gentamicine, IZ^b^ (1 mg/mL)	19.5	21.5	22.5	20.5
MIC (% *v*/*v*)	0.0625	0.125	0.5	1
MBC (% *v*/*v*)	0.25	1	2	4

All values in this table represent mean ± SD (n = 3).

^a^ Used volume for disc diffusion method.

^b^ Diameter of inhibition zone (mm).

**TABLE 4:** Evaluation of the antifungal activity of LPEO.

Fungal strain	*P. digitatum*	*A. niger*	*C. glabrata*	*R. glutinis*
15 µL^a^ of Essential oil, IZ^b^	23.00 ± 0.20	23.20 ± 0.4	25.30 ± 0.30	21.60 ± 0.50
15 µL^a^ Cycloheximide, IZ^b^ (1 mg/mL)	19.5	21.5	22.5	20.5
MIC (% *v*/*v*)	1	1	0.5	2
MBC (% *v*/*v*)	4	0.25	2	4

All values in this table represent mean ± SD (n = 3).

^a^ used volume for disc diffusion method.

b diameter of inhibition zone (mm).

The results of our study demonstrate that the LPEO exhibits notable antibacterial and antifungal activities. Analysis of its composition revealed the significant presence of compounds such as carvacrol and thymol, well-known for their antimicrobial properties (Marchese et al., 2016; Zhang et al., 2018; Najafloo et al., 2020). However, it is crucial to emphasize that the effectiveness of the essential oil cannot be solely attributed to a single compound. This observation suggests that the synergy among various compounds, including those present in minor quantities, may play a pivotal role in the expression of this antimicrobial activity. A comparative analysis was conducted with other Lavandula species, corroborating the presented results. Tofah et al. affirmed the notable antimicrobial activity of *L. multifida L.* (Tofah et al., 2022). Furthermore, Blažeković et al. highlighted the remarkable antimicrobial properties of the essential oil from *Lavandula × intermedia* “*Budrovka*” (Blažeković et al., 2018). Collectively, these findings substantiate the remarkable antimicrobial activity of the LPEO, positioning it as a promising and sustainable source for natural alternatives against a spectrum of microbial strains.

### Anti-gout, antidiabetic activity

The present study evaluates the anti-gout and the antidiabetic activity of LPEO, on three enzymes, namely, xanthine oxidase, α-amylase and α-glucosidase. The IC50 of LPEO for xanthine oxidase inhibition was determined to be 26.48 ± 0.90 μg/mL. By way of contrast, allopurinol, a prototypical inhibitor of xanthine oxidase (XO), has served as a fundamental element in the clinical treatment of gout and conditions linked to elevated uric acid levels for numerous decades, and it was used as a positive control in the present study. Allopurinol showed a slightly higher inhibition with an IC50 of 23.34 ± 0.09 μg/mL. These results indicate significant activity of the EO in inhibiting xanthine oxidase, an enzyme catalyzing the degradation of hypoxanthine to uric acid under conditions of high ATP demand and oxygen deficiency (Schmidt et al., 2020). Detailed results of these assays are presented in Table 5. Previous studies have suggested that xanthine oxidase inhibition may have a beneficial impact on endothelial function, often compromised in individuals with diabetes (Desco et al., 2002). Regarding α-amylase inhibition (AAI), the IC50 of the EO was determined to be 31.56 ± 0.46 μg/mL, while the positive control acarbose demonstrated similar activity with an IC50 of 35.48 ± 0.69 μg/mL. These results suggest a notable capacity of the EO to inhibit α-amylase, an enzyme involved in carbohydrate digestion (Rodríguez-Viera et al., 2016). α-amylase, responsible for breaking down starch into simple sugars, has been correlated with glucose metabolism, and previous studies suggest that a low α-amylase level increases the risk of metabolic dysfunction, insulin resistance, and type 2 diabetes (Nakajima et al., 2011). Concerning α-glucosidase inhibition (AGI), the IC_50_ of the EO increased to 58.47 ± 2.35 μg/mL, while the positive control acarbose revealed a more pronounced inhibition with an IC50 of 65.41 ± 2.10 μg/mL. These results indicate significant activity of the EO in inhibiting α-glucosidase, suggesting potential benefits in regulating postprandial glucose release. α-Glucosidase inhibitors, a category of oral antidiabetic drugs, act by impeding carbohydrate digestion, thereby reducing blood glucose levels and potentially preventing or delaying the onset of type 2 diabetes and its complications in individuals at risk of developing diabetes (Bhatia et al., 2019). LPEO has demonstrated promising antidiabetic properties, hypothetically attributed to the abundance of terpene molecules such as carvacrol, whose antidiabetic activity has been demonstrated in several studies (Imran et al., 2022; Aljelehawy et al., 2023a), as well as thymol, a bioactive molecule with notable antidiabetic activities (Aljelehawy et al., 2023b). Camphene has also exhibited notable antidiabetic activity (Hachlafi et al., 2023). The combined presence of these three molecules could thus be the main cause of this antidiabetic activity. Regarding the distinctiveness of L. pinnata indigenous to the Oriental region (Morocco), and the lack of information on this plant in the existing literature, the intricate nature of its chemical composition presents difficulties in directly comparing it with counterparts from other regions. To strengthen the validity of our results, a comparative analysis was undertaken with Lavandula stoechas, supported by an *in vitro* study done by El Hachlafi et al. (2023), thus confirming our observations of antidiabetic activity. These results pave the way for the potential use of LPEO as a natural alternative for antidiabetic applications, thereby expanding the range of possibilities in the field of health.

**TABLE 5:** α-amylase, Xanthine Oxidase and α-glucosidase inhibitory activities of LPEO in terms of IC_50_ values.

Essential oil/Positive control	IC_50_ (µg/mL), ±SD			
	XO inhibition	AAI activity	AGI activity	
LPEO	26.48 ± 0.90	31.56 ± 0.46	58.47 ± 2.35	
Allopurinol^a^	23.34 ± 0.09	-	-	
Acarbose^a^	-	35.48 ± 0.69	65.41 ± 2.10	

^a^ Positive controls. Values are means ± SD (n = 3).

#### Tyrosinase inhibition assay

The promising findings about LPEO’s anti-tyrosinase activity carry paramount implications for cutaneous protection. Tyrosinase, an indispensable catalyst in melanin biosynthesis, affects the transformation of tyrosine into the pigment governing the coloring of skin, hair and eyes (Bouyahya et al., 2021). The meticulous regulation of this enzyme assumes a pivotal role in governing pigmentation and mitigating the harmful consequences of UV radiation, encompassing cellular detriment, premature senescence, and the heightened predisposition to cutaneous malignancies (Bouyahya et al., 2019). These revelations proffer auspicious trajectories for the innovation of avant-garde dermatological formulations. The outcomes from assessing LPEO, specifically its antityrosinase activity, yield encouraging prospects. The determined IC50 for LPEO stands at 29.11 ± 0.08 mg/mL, whereas the comparative control, Quercetin, manifests an IC50 of 22.15 ± 0.12 mg/mL. A lower IC50 value connotes heightened efficacy in enzyme inhibition (Table 6). Therefore, despite the noticeable anti-tyrosinase activity observed in LPEO, it seems to demonstrate a level of effectiveness lower than that of quercetin, the specified control compound. This notable anti-tyrosinase activity is likely attributed to the significant presence of carvacrol in LPEO, a hypothesis supported by El Khoury et al.'s (El Khoury et al., 2019), confirmation of carvacrol’s considerable anti-tyrosinase activity.

**TABLE 6:** *In vitro* dermatoprotective activity using Tyrosinase inhibition assay.

Assay	LPEO	Control (quercetin)
	IC_50_ (mg/mL)^a^	
Tyrosinase	29.11 ± 0.08	22.15 ± 0.12

^a^ Values are mean ± SD (n = 3).

Given the exclusive nature of our investigation in appraising LPEO’s anti-tyrosinase activity, a compelling exigency arises to juxtapose it against analogous specimens within the same taxonomic classification. By way of illustration, the EO derived from Lavandula officinalis, lauded for its dermatoprotective efficacy by Cheraif et al. (2020), serves as a prospective benchmark. These findings portend the plausible efficacy of LPEO as a dermatoprotective agent, thereby instigating further inquiries aimed at ratifying this conjecture and elucidating the intricate mechanistic underpinnings of these dermatologically relevant attributes.

### Anticancer activity: cell viability by MTT assay

**FIGURE 2:**
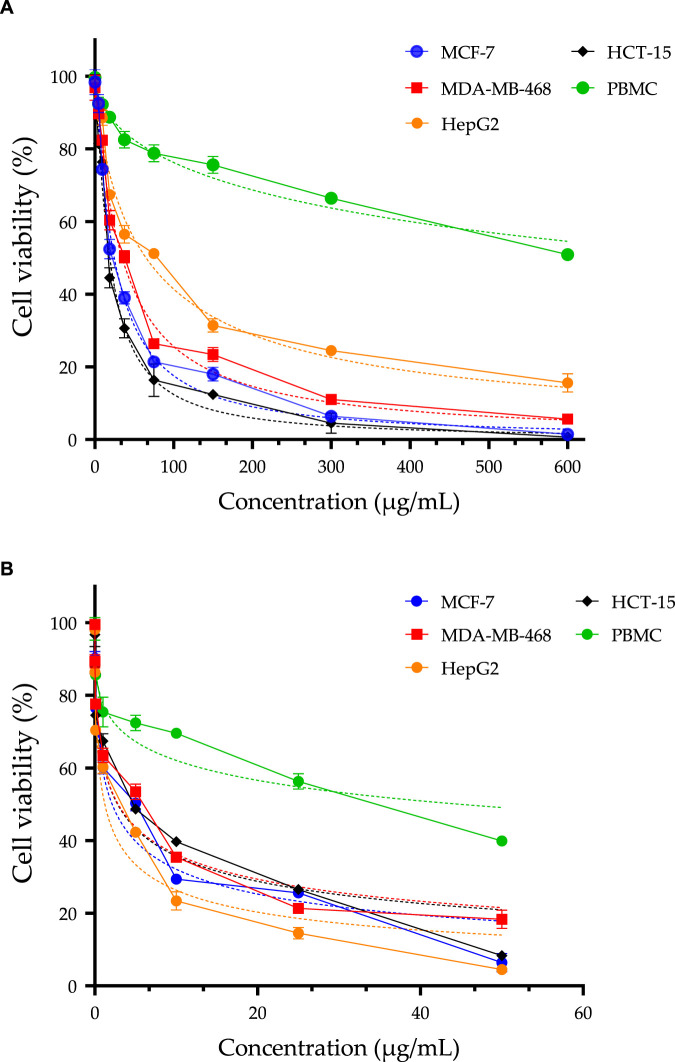
MTT assay was used to assess the cell viability of MCF-7, MDA-MB-468, HepG2, HCT-15, and PBMC cells following a 72-h treatment with LPEO **(A)**, and doxorubicin [positive control, **(B)**]. The IC_50_ values are presented as means ± SD and are derived from three independent studies.

The essential oil of *L. pinnata* was investigated for its cytotoxic activity against four cancer cell lines (MCF-7, MDA-MB-468, HepG2, and HCT-15) and a normal cell line (PBMC) using the MTT assay. Cisplatin, a widely utilized chemotherapy agent (Fennell et al., 2016), acts by forming covalent bonds with cellular DNA, inducing lesions and thereby inhibiting cell division (Chen et al., 2014). This property renders it a crucial antineoplastic agent in the treatment of various cancer types. As a reference drug, cisplatin was employed to compare and assess the cytotoxic efficacy of LPEO. IC50 values and the Selectivity Index (SI) were calculated and are presented in Table 7 and Figure 2. The results demonstrated that LPEO exhibits moderate cytotoxic activity against cancer cells, with IC_50_ values ranging from 18.87 to 64.92 μg/mL, in comparison to cisplatin, which has IC_50_ values ranging from 1.22 to 2.87 μg/mL. LPEO displayed low toxicity towards normal cells, with an IC_50_ value of 837.20 μg/mL, significantly higher than that of cisplatin (44.88 μg/mL). SI values indicate the degree of selectivity of LPEO and cisplatin for cancer cells compared to normal cells. A higher SI suggests greater selectivity. The results revealed that LPEO has a higher SI than cisplatin for MCF-7 and HCT-15 cell lines, indicating that LPEO is more selective for these types of cancer than cisplatin. Conversely, LPEO has a lower SI than cisplatin for MDA-MB-468 and HepG2 cell lines, suggesting that LPEO is less selective for these types of cancer than cisplatin. These findings align with prior studies reporting the cytotoxic activity of specific compounds within LPEO, such as carvacrol and thymol (Aydın et al., 2017; Sharifi Rad et al., 2018; Ahmad et al., 2021; Bansal et al., 2022). Additionally, due to the lack of specific studies on LPEO, a comparison was made with another study conducted by Fahmy et al. on another lavender species, demonstrating significant cytotoxic effects of *Lavandula officinalis* essential oil on HepG2 and A549 cell lines, with an IC50 values of 67.8 and 12 μg/mL, respectively (Fahmy et al., 2022). These results indicate that LPEO exhibits moderate cytotoxic activity against breast (MCF-7 and MDA-MB-468), liver (HepG2), and colon (HCT-15) cancer cells, with low toxicity towards normal cells (PBMC). LPEO proves to be more selective than cisplatin for breast (MCF-7) and colon (HCT-15) cancer cells but less selective than cisplatin for breast (MDA-MB-468) and liver (HepG2) cancer cells.

**TABLE 7:** IC_50_ values, and Selectivity indices of LPEO on cancer cell lines (MCF-7, MDA-MB-468, HepG2, and HCT-15).

Cell lines	IC_50_ values (µg/mL)		Selectivity Index (SI)	
	LPEO	Cisplatin	LPEO	Cisplatin
MCF-7	24.62 ± 0.98	2.13 ± 0.07	34.00 ± 1.43	21.53 ± 1.10
MDA-MB-468	34.61 ± 2.32	2.87 ± 0.29	24.21 ± 0.65	15.62 ± 1.46
HepG2	64.92 ± 4.29	1.22 ± 0.09	12.89 ± 0.17	36.72 ± 2.44
HCT-15	18.87 ± 1.27	2.79 ± 0.13	44.32 ± 0.04	16.08 ± 1.50
PBMC	837.20 ± 9.51	44.88 ± 2.51	-	-

Data are obtained from three independent experiments and expressed as means ± SD. * Selectivity index = (IC_50_ of LPEO, on PBMC, cells/IC_50_ of LPEO, on tumor cells).

## Conclusion

In conclusion, the comprehensive assessment of *L. pinnata* essential oil (LPEO) has revealed its diverse and promising biological activities. Chemical analysis using gas chromatography-mass spectrometry (GC-MS) has identified a complex composition, rich in bioactive compounds, which contribute to LPEO’s multifaceted therapeutic potential. LPEO has demonstrated remarkable antioxidant capabilities, as evidenced by its high antiradical activity and significant total antioxidant capacity. Its broad-spectrum antibacterial and antifungal activities, as well as the inhibition of key enzymes such as xanthine oxidase, α-amylase, α-glucosidase, and tyrosinase, highlight its potential in managing oxidative stress, postprandial glucose levels, and skin-related concerns. Furthermore, LPEO has shown moderate cytotoxic activity against certain cancer cell lines, with selectivity towards cancer cells over normal peripheral blood mononuclear cells (PBMC), particularly notable against the non-metastatic breast cancer cell line (MCF-7) and colorectal adenocarcinoma cells (HCT-15).

Future research directions include expanding the scope to investigate other plant species with similar or complementary bioactive profiles to LPEO. This expansion could potentially unveil new therapeutic agents and broaden the applicability of the findings. The limitations of the current study, primarily the *in vitro* nature of the assessments, may not fully predict the *in vivo* efficacy and safety profile of LPEO. Additionally, the complexity of LPEO’s composition necessitates further isolation and characterization of individual compounds to understand their specific contributions to the observed biological activities. Future studies will focus on *in vivo* evaluations to confirm the therapeutic potential of LPEO in a physiological context. Investigations into the synergistic effects of LPEO’s constituents and exploration of formulation strategies to enhance its bioavailability and efficacy are also planned. Through these approaches, the intricate mechanisms of action will be elucidated, paving the way for clinical applications of LPEO and other natural therapeutic agents.

## Data Availability

The raw data supporting the conclusion of this article will be made available by the authors, without undue reservation.
