# Supplementary material for: Exploring the multifaceted bioactivities of Lavandula pinnata L. essential oil: promising pharmacological activities
Source: Front Chem. 2024 Apr 8;12:1383731. doi: 10.3389/fchem.2024.1383731 (PMC11041020; doi:10.3389/fchem.2024.1383731)

Exploring the Multifaceted Bioactivities of *Lavandula pinnata L.* Essential Oil: promising pharmacological activities by use of in *vitro and in silico* approaches

Mounir Haddou^1,2*^, Amine Elbouzidi^1,3^, Mohamed Taibi^1,2^, Abdellah Baraich^4^, El Hassania Loukili^3^, Reda Bellaouchi^4^, Ennouaamane Saalaoui^4^, Abdeslam Asehraou^4^, Ahmad Mohammad Salamatullah^5^, Mohammed Bourhia^6*^, Hiba-Allah Nafidi^7^, Mohamed Addi^1^, Bouchra El Guerrouj^1,2^, Khalid Chaabane^1^

**Figure S1 :** Inhibition zones observed for various bacterial strains. **1:** *Staphylococcus aureus*, **2:** *Micrococcus luteus*, **3:** *Escherichia coli*, **4:** *Pseudomonas aeruginosa.*


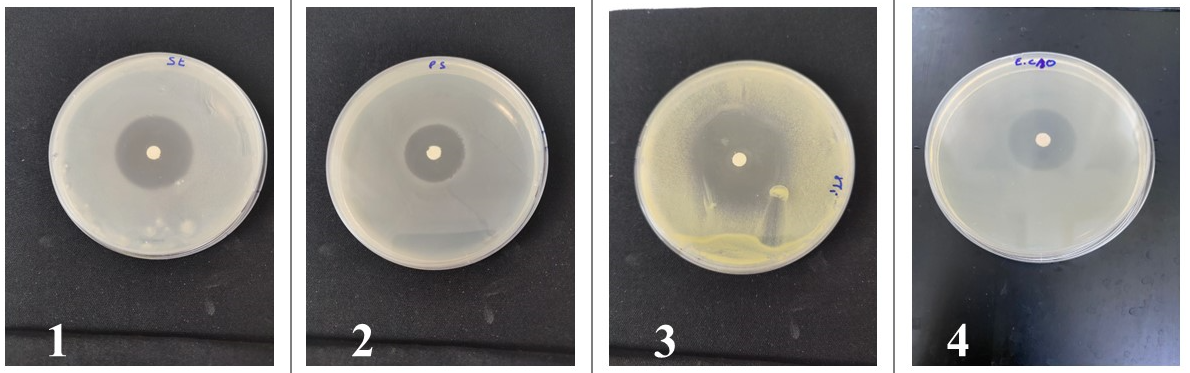


**Figure S2 :** Inhibition zones observed for various fungal strains. **(a)** *Penicillium digitatum***, (b)** *Aspergillus niger***, (c)** *Candida glabrata***, (d)** *Rhodotorula glutinis***.**
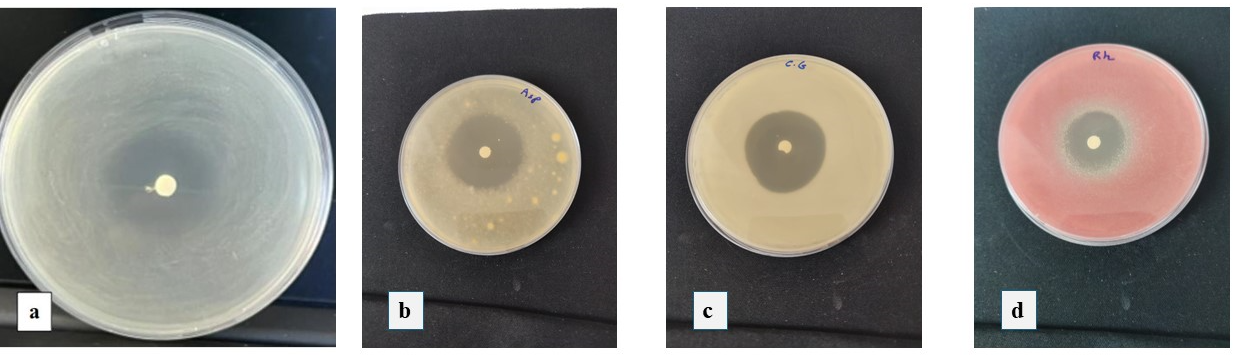

Supplement: Supplementary file 1 [file DataSheet1.docx]
